# Effects of prolonged vibration to the flexor carpi radialis muscle on intracortical excitability

**DOI:** 10.1038/s41598-024-59255-5

**Published:** 2024-04-11

**Authors:** Clara Pfenninger, Narimane Zeghoudi, Mathilde Fiona Bertrand, Thomas Lapole

**Affiliations:** Laboratoire Interuniversitaire de Biologie de la Motricité, Université Jean Monnet Saint-Etienne, Lyon 1, Université Savoie Mont-Blanc, 42023 Saint-Étienne, France

**Keywords:** Neuroscience, Physiology

## Abstract

Prolonged local vibration (LV) can induce neurophysiological adaptations thought to be related to long-term potentiation or depression. Yet, how changes in intracortical excitability may be involved remains to be further investigated as previous studies reported equivocal results. We therefore investigated the effects of 30 min of LV applied to the right flexor carpi radialis muscle (FCR) on both short-interval intracortical inhibition (SICI) and intracortical facilitation (ICF). SICI and ICF were measured through transcranial magnetic stimulation before and immediately after 30 min of FCR LV (vibration condition) or 30 min of rest (control condition). Measurements were performed during a low-intensity contraction (n = 17) or at rest (n = 7). No significant SICI nor ICF modulations were observed, whether measured during isometric contractions or at rest (p = 0.2). Yet, we observed an increase in inter-individual variability for post measurements after LV. In conclusion, while intracortical excitability was not significantly modulated after LV, increased inter-variability observed after LV may suggest the possibility of divergent responses to prolonged LV exposure.

## Introduction

Local vibration (LV), applied to a muscle or its tendon, induces repeated Ia afferents discharge^1^ that project at both spinal and cortical levels inducing neuromuscular adaptations^2^. Recently, prolonged LV (≈ 30–60 min) has emerged as a new rehabilitation method, the repetition of LV sessions showing the potential to improve motor function on the long-term^3^. Such motor improvements could be the result of repeated sensory stimulation inducing synaptic plasticity along proprioceptive pathways and within sensorimotor cortex areas^4^. It is further speculated that LV could induce long-term potentiation or depression, as suggested by acute and long-lasting effects of LV on intracortical excitability^4^. Yet, further studies are required to better understand intracortical effect of LV as previous studies reported equivocal results^2,4^.

Intracortical excitability can be investigated through paired-pulse transcranial magnetic stimulation (TMS). More precisely, the activation of intracortical inhibitory circuits mediated by gamma-aminobutyric acid type A (GABA_A_) occurs when a subthreshold conditioning pulse is administered before a suprathreshold test stimulus with a short interval of 1–5 ms. The so-called short-interval intracortical inhibition (SICI) can then be evidenced through a decrease in the amplitude of the conditioned motor-evoked potentials (MEP) when compared to the non-conditioned one. On the other hand, the activation of intracortical facilitatory circuits mediated by glutamate occurs when using the same paired-pulse TMS paradigm at a longer interval of 10–15 ms, the resulting intracortical facilitation (ICF) being evidenced by an increase in the conditioned MEP amplitude^5^.

During LV, SICI has been reported to decrease^6–10^ and a small increase or an absence of ICF modulation has been reported^6–8^. It has been suggested that the LV-induced proprioceptive inputs targeting cortical areas could have a direct effect on intra-cortical inhibition and facilitation. Specifically, by reducing SICI and to a lesser extent by increasing ICF, this balance shifts towards an increase in intracortical excitability^7^, likely explaining, at least partly, increased corticospinal excitability (i.e., unconditioned MEP amplitude) during LV^11,12^.

While there are evidence of corticospinal modulations after prolonged periods of afferent stimulation through peripheral nerve stimulation^13^ results are more equivocal after prolonged LV^14–17^. This is likely because prolonged LV is known to decrease motoneuron excitability, as suggested by LV-decrease in responses to corticospinal tract electrical stimulation^14,16,17^. Increased cortical excitability would be therefore hidden when measuring MEP amplitude known to depend on both spinal and cortical excitability. Increased cortical excitability after prolonged LV was accordingly reported when MEP amplitude was interpreted in light of changes in motoneuron excitability^14,16,17^. There also exist studies investigating changes in intracortical excitability after prolonged LV. A decrease in SICI, together with an increase in ICF, has been reported after 20 min of whole hand vibration, suggesting short term plasticity in intracortical circuits^18^. However, results about intracortical excitability after prolonged LV are equivocal, the majority of studies showing an absence of modulation immediately after LV^9,10,19^. While differences in vibration characteristics could explain discrepancies between studies, the way intracortical excitability was investigated in the aforementioned studies could also explain the lack of consensual results. For instance, findings of increased cortical excitability after prolonged LV have always been observed when measurements were performed during a voluntary contraction^14,16,17^. When now considering intracortical excitability, measurements have to our knowledge only been performed at rest^9,10,19^, so that it remains hazardous to relate changes in cortical excitability observed after prolonged LV by potential changes in intracortical excitability. Similarly, electroencephalographic (EEG) studies revealed increased cortical activity after prolonged LV, likely related to increased cortical excitability^20^, when measurements were performed during an isometric contraction^21^ but not when recordings were performed during resting state^22^.

While previous research considered the effect of prolonged LV on measures of intracortical excitability recorded during resting state, we used paired-pulse magnetic stimulation during isometric contractions to assess the effects of 30 min of LV applied to the right flexor carpi radialis muscle (FCR) on SICI and ICF. We hypothesised a decrease in SICI and an increase in ICF after prolonged LV. A subgroup of participants performed additional sessions to measure SICI and ICF during resting state after prolonged LV, assuming absence of modulation.

## Materials and methods

### Participants

20 healthy participants (10 men and 10 women; age: 25 ± 3.7 years; stature: 168.3 ± 10.8 cm; mass: 65.5 ± 9.2 kg) were included in the experiment. All participants were free from neurological disease and musculoskeletal injury and had no contraindications to TMS^23^. The study was approved by the institutional ethics committee (Comité de Protection des Personnes SudEst I; 1408208) and was conformed to the *Declaration of Helsinki*, except for registration in a database. Written informed consent was obtained from each participant prior to the study begin.

### Experimental design

**Figure 1:**
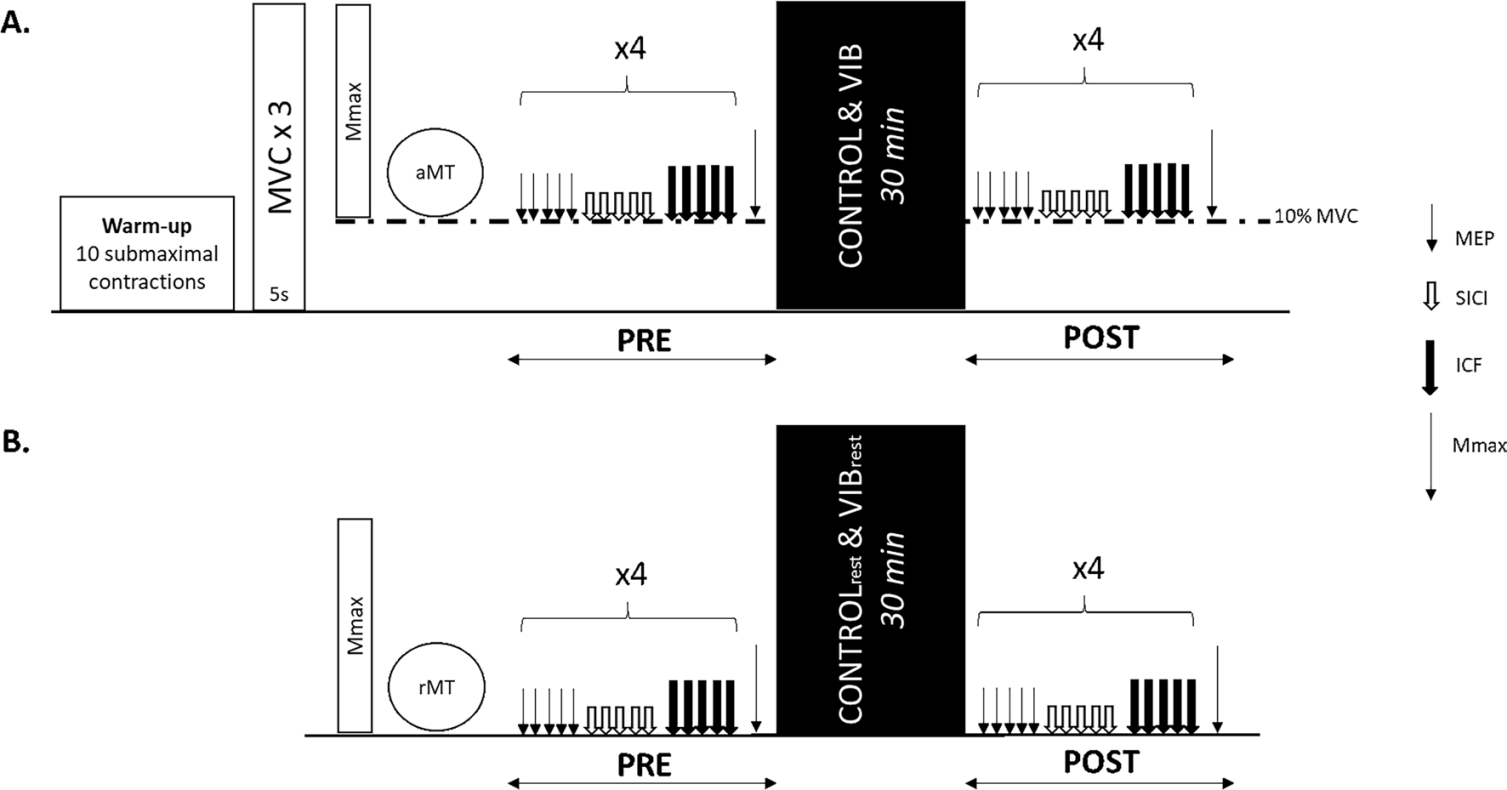
Schematic overview of the experimental protocol performed before (PRE) and after (POST) each condition. (**A**) Measurements were performed during a low level of isometric contraction at 10% MVC (n = 20). (**B**) Measurements were performed at rest (n = 7). MVC, maximal voluntary contraction; aMT, active motor threshold; rMT, resting motor threshold; MEP, motor evoked potential; SICI, short-interval intracortical inhibition; ICF, intracortical facilitation.

Participants visited the laboratory for two randomized sessions: a control condition (CONTROL) and a vibration condition (VIB) performed at the same time of the day with at least two to seven days between sessions. As illustrated in Fig. 1, experimental sessions comprised corticospinal and intracortical excitability assessments on the right flexor carpi radialis (FCR) muscle before (PRE) and after (POST) each condition. Measurements included the recordings of motor evoked potentials (MEPs; measure of corticospinal excitability), short-interval intracortical inhibition (SICI), intracortical facilitation (ICF) and M-wave (measure of muscle fibers excitability). All the measurements were performed during a low-intensity wrist flexion contraction corresponding to 10% of maximal voluntary contraction (MVC). A subset of 7 participants participated in two additional sessions (CONTROLr and VIBr, respectively), the same measurements being performed on the relaxed FCR.

### Experimental procedures

For VIB and CONTROL sessions, the baseline measurements (PRE) began with a standardized warm up of ten submaximal isometric contractions, gradually increasing the force produced to approach the maximum wrist flexion force. Then, participants performed two 5-s MVCs separated by 60 s of rest. If necessary, they performed additional MVC until the highest MVC did not further increase (no more than 5% variation between the two last MVCs). During MVCs, participants were instructed to contract as hard as possible and were verbally encouraged by the experimenter. Then a target was set at 10% of this MVC, participants being asked to reach the target and maintain this force level during measurements. First, FCR maximal M-wave (Mmax) was measured during the ongoing contraction before optimal stimulation intensities for TMS were determined. Once all appropriate intensities had been determined, baseline measurements were performed. This consisted in four sets of five MEP, five SICI and five ICF responses. At the end of the four sets, one Mmax was recorded. Each evoked potential was performed during a single contraction, lasting approximatively 3 s, with 5–7 s of rest between trials. The order of stimulations (i.e., MEP, SICI, ICF, M-wave) was always the same (i.e., no randomization). The same measurements, keeping the intensity of stimulation defined at PRE, were performed at POST.

For the 7 participants performing the two additional sessions (CONTROLr and VIBr), experimental procedures were the same except that measurements were performed at rest. Therefore, there was no force measurement.

### Conditions

For the vibration condition (VIB & VIBr), LV (100 Hz with an amplitude of 1 mm; VB 115, Techno Concept, Mane, France) was applied to the muscle belly of the relaxed right FCR installed in the ergometer. The application lasted 10 min and was repeated three times with an interval of one min as described in previous studies^16,24,25^. For the control condition (CONTROL & CONTROLr), the arm of the participants was positioned in the ergometer and participants were asked to remain fully relaxed for 30 min.

### Instrumentations

#### Force recording

**Figure 2:**
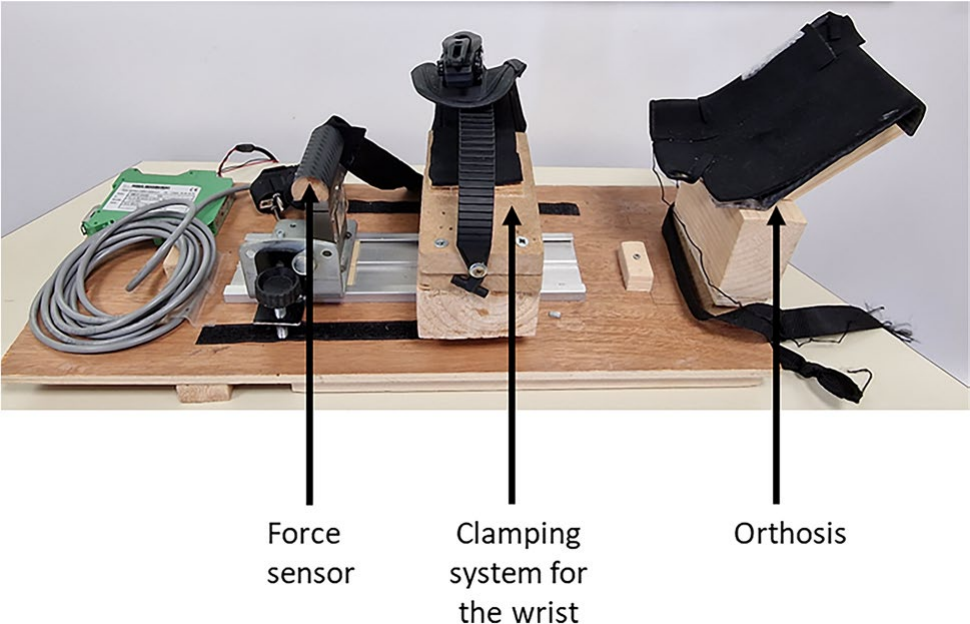
Illustration of the custom-built ergometer.

Voluntary isometric wrist flexion force was recorded with a custom-built ergometer (Fig. 2). The same ergometer was used for positioning the arm during the vibration or relaxation period. The right arm of the participants was blocked in an orthosis with an elbow angle of 120°, a shoulder abduction of 20°, and no shoulder flexion. The forearm was locked in a pronation position with a clamping system at the wrist and a force sensor positioned in the palm of the hand to measure the strength in wrist flexion. This position was maintained throughout the entire session.

#### Electromyography (EMG)

Participants were first prepared by shaving, gently abrading the skin, and then cleaning it with isopropyl alcohol. EMG of the FCR muscle was recorded with a pair of self-adhesive surface electrodes (Meditrace 100, Covidien, Mansfield, MA) placed in a belly-tendon montage. The reference was placed on the ulna styloid process. Signal was bandpass filtered (10–500 Hz), amplified by bio-amplifier (ML138, ADInstruments; common mode rejection ration = 85 db, gain = 5000) and analogue-to-digitally converted at a sampling rate of 2000 Hz by Powerlab system (16/30-ML880/P, ADInstruments, Bella Vista, Australia). All data were analysed offline using Labchart 8 software (ADInstruments).

#### Peripheral nerve stimulation

The right median nerve was stimulated by a single rectangular electrical stimulus with a duration of 0.1 ms and a maximum output voltage of 400 V (DS7A, Digitimer, Welwyn Garden City, Hertfordshire, UK) delivered through a bipolar bar stimulating electrode with 30 mm anode–cathode spacing (Bipolar Felt Pad Stimulating Electrode Part Number E. SB020/4 mm, Digitimer) placed at the cubital fossa. Electrical stimuli were first administered at 5 mA and then were increased by 5-mA steps until the maximal M-wave amplitude (Mmax) was obtained. The optimal intensity was then increased by 20% to ensure supramaximal stimulation. Mmax was measured on the right FCR during the 10% MVC previously defined for CONTROL and VIB sessions. For CONTROLr and VIBr sessions, Mmax was measured on the right FCR while at rest.

#### Transcranial magnetic stimulation

Single- and paired pulse TMS were delivered over the left motor cortex via a figure-of-eight coil using a BiStim unit and two Magstim 200^2^ stimulator (Magstim Co., Ltd., Whitland, United Kingdom). The coil was positioned tangentially to the scalp (at a 45° angle to the midline) to induce a posterio-anterior current. The optimal coil placement was determined as the position that elicited the largest MEP in the FCR with an intensity of 50% of the maximal stimulator output (MSO). Starting at 50% MSO is conventionally used in our laboratory^16,26^ because it is safe and well-tolerated by participants. If a participant however presented a threshold above 50% MSO (i.e. no MEPs were visible at 50% MSO after having tested at least 5 different sites of stimulation), we increased stimulation intensity to 60% MSO before re-evaluating the hotspot. A further increase to 70% MSO was performed if no visible and consistent MEPs were observed at 60% MSO. Once identified, this position was marked directly on a swimming pool cap worn by participants to ensure consistent positioning throughout the experiment.

For CONTROL and VIB sessions, the stimulator intensity was based on the active motor threshold (aMT) previously established during a 10% MVC contraction. In order to determine aMT, we initiated the procedure at 50% of the MSO and decreased the intensity in 5% increments until we failed to elicit 3 MEPs out of 5 trials exceeding 200 μV. Subsequently, we increased the intensity in 1% increments until we were able to consistently evoke 3 MEPs out of 5 trials with amplitudes surpassing the predefined threshold. Then, the intensity for non-conditioned (i.e., single pulse TMS) and conditioned (i.e., paired pulse TMS) MEPs was set at 120% of this aMT, and the intensity for the conditioning pulse was set at 70% of aMT^25,27^. Individual values of aMT are presented in supplementary data (Table 1). Inter-stimulus interval was set at 2 ms for SICI and 10ms for ICF^28–30^.

For CONTROLr and VIBr sessions, the stimulator intensity was based on the resting motor threshold (rMT). In order to determine rMT, we initiated the procedure at 50% of the MSO and decreased the intensity in 5% increments until we failed to elicit 3 MEPs out of 5 trials exceeding 50 µV. Subsequently, we increased the intensity in 1% increments until we were able to consistently evoke 3 MEPs out of 5 trials with amplitudes surpassing the predefined threshold. Then, the intensity for non-conditioned (i.e., single pulse TMS) and conditioned (i.e., paired pulse TMS) MEPs was set at 120% of this rMT, and the intensity for the conditioning pulse was set at 70% of rMT^25,27^. Individual values of rMT are presented in supplementary data (Table 1). The same inter-stimuli intervals as presented above were used for SICI and ICF.

### Data analysis

At each time point (i.e. PRE and POST), the mean peak-to-peak amplitudes of the 20 MEPs, SICI and ICF responses were used for statistical analysis. For each sessions, the EMG background activity was measured as the root mean square (RMS_EMG_) calculated from − 0.05 to − 0.1 s prior to each TMS stimulus, averaged for each time point and expressed in percentage of the corresponding Mmax amplitude. MEPs were expressed in percentage of their corresponding Mmax amplitude. To quantify SICI and ICF, the ratio of the averaged conditioned MEP responses was expressed as a percentage of the mean unconditioned MEP amplitude. Specifically, SICI was calculated as follows: 100%−(mean conditioned MEP/mean unconditioned MEP). ICF was calculated as follows: (mean conditioned MEP/mean unconditioned MEP)−100%.

### Statistical analyses

The analyses were conducted using R Studio (version 1.3.1093). In all statistical tests, a significance level of p < 0.05 was employed. The data were presented in terms of mean values ± standard deviations (SD). Normal distribution tested with Shapiro–Wilk test failed and Wilcoxon Tests were used to compare MEP, SICI and ICF responses at PRE between CONTROL and VIB on one hand, and between CONTROLr and VIBr on the other hand.

To ascertain the relationship between intracortical excitability, condition, and time, a generalized mixed model analysis was carried out using the *glmmTMB* statistical framework^31^. The model included condition and time (along with their interaction term) as fixed effects. Subject intercepts were incorporated as random effects. Visual examination of residual plots did not reveal any obvious deviations from homoscedasticity or normality.

## Results

For CONTROL and VIB sessions, data are presented for 17 participants as three participants were excluded due to an absence of SICI at PRE.

### RMS

Muscular activity was not modified across time point between CONTROL (PRE: 0.42 ± 0.15%, POST: 0.43 ± 0.15%) and VIB conditions (PRE: 0.42 ± 0.12%, POST: 0.41 ± 0.12%) (p = 0.6). Similar results were observed for CONTROLr (PRE: 0.02 ± 0.02%, POST: 0.02 ± 0.02%) and VIBr (PRE: 0.02 ± 0.02%, POST: 0.02 ± 0.02%) (p = 0.5).

### MEP responses

**Figure 3:**
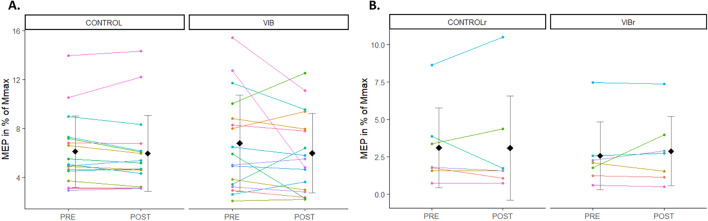
Mean ± SD and individual MEP amplitudes in % of Mmax. Each dot represents the value of a participant. (**A**) PRE and POST values are represented for CONTROL and VIB conditions. (**B**) PRE and POST values are represented for CONTROLr and VIBr conditions. Participants who performed the four testing sessions are displayed with the same colour across panels (**A**) and (**B**).

MEP amplitudes were similar at PRE between CONTROL and VIB conditions (V = 41, p = 0.10), as well as between CONTROLr and VIBr conditions (V = 23, p = 0.16). MEP amplitudes were similar across time points between CONTROL (PRE: 6.11 ± 2.92%, POST: 5.96 ± 3.12%) and VIB conditions (PRE: 6.79 ± 3.94%, POST: 5.98 ± 3.26%) (p = 0.4) (Fig. 3A). Similar results were observed for CONTROLr (PRE: 3.10 ± 2.67%, POST: 3.07 ± 3.48%) and VIBr (PRE: 2.56 ± 2.27%, POST: 2.87 ± 2.31%) (p = 0.6) (Fig. 3B).

### SICI responses

**Figure 4:**
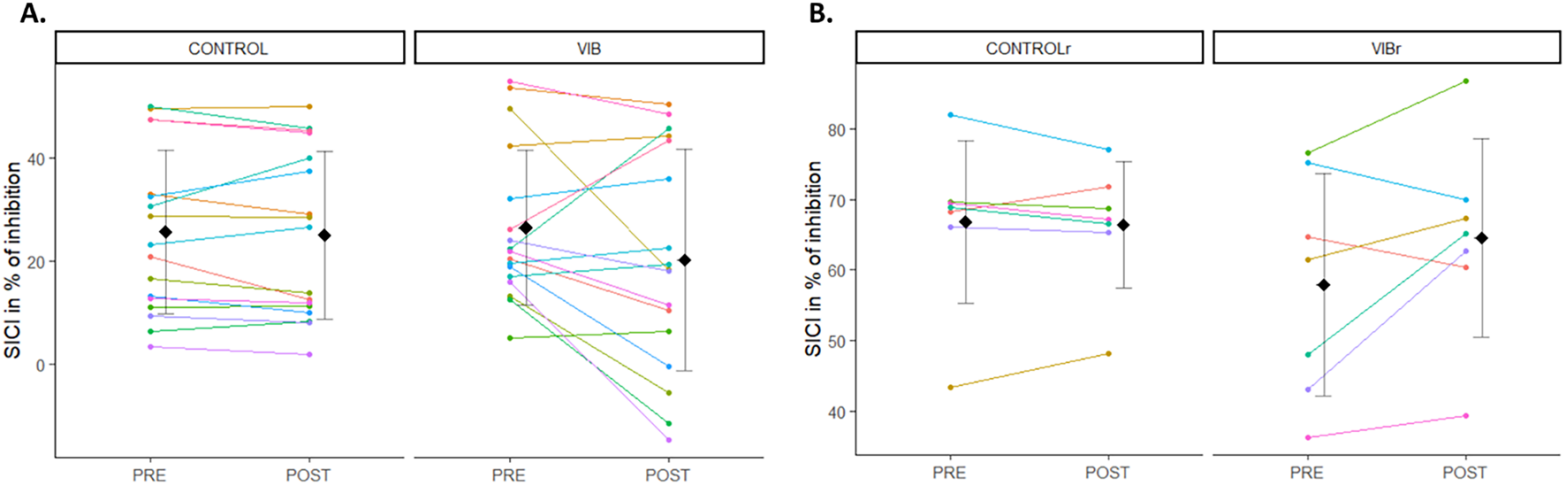
Mean ± SD and individual SICI responses in % of inhibition. Each dot represents the value of a participant. (**A**) PRE and POST values are represented for CONTROL and VIB conditions. (**B**) PRE and POST values are represented for CONTROLr and VIBr conditions. Participants who performed the four testing sessions are displayed with the same colour across panels (**A**) and (**B**).

SICI was similar at PRE between CONTROL and VIB conditions (V = 84, p = 0.75), as well as between CONTROLr and VIBr conditions (V = 7, p = 0.30). SICI were similar across time points between CONTROL (PRE: 25.65 ± 15.83%, POST: 25.02 ± 16.22%) and VIB conditions (PRE: 26.44 ± 14.91%, POST: 20.17 ± 21.45%) (p = 0.2) (Fig. 4A). Similar results were observed for CONTROLr (PRE: 66.80 ± 11.53%, POST: 66.39 ± 8.98%) and VIBr (PRE: 57.91 ± 15.74%, POST: 64.55 ± 14.04%) (p = 0.3) (Fig. 4B).

### ICF responses

**Figure 5:**
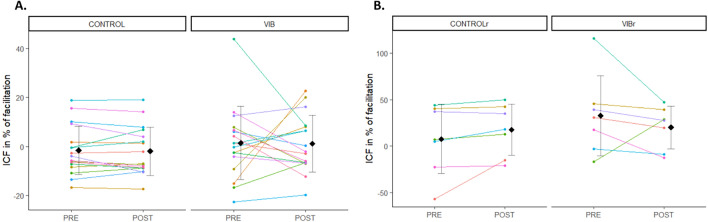
Mean ± SD and individual ICF responses in % of facilitation. Each dot represents the value of a participant. (**A**) PRE and POST values are represented for CONTROL and VIB conditions. (**B**) PRE and POST values are represented for CONTROLr and VIBr conditions. Participants who performed the four testing sessions are displayed with the same colour across panels (**A**) and (**B**).

ICF was similar at PRE between CONTROL and VIB conditions (V = 61, p = 0.49), as well as between CONTROLr and VIBr conditions (V = 7, p = 0.30). ICF were similar across time points between CONTROL (PRE: − 1.60 ± 9.91%, POST: − 1.95 ± 9.82%) and VIB conditions (PRE: 1.44 ± 14.86%, POST: 1.09 ± 11.58%) (p = 0.9) (Fig. 5A). Similar results were observed for CONTROLr (PRE: 7.63 ± 37.32%, POST: 17.38 ± 27.56%) and VIBr (PRE: 32.70 ± 43.05%, POST: 20.14 ± 22.97%) (p = 0.1) (Fig. 5B).

## Discussion

The objective of our study was to investigate intracortical excitability modulations after prolonged LV applied to the relaxed FCR muscle. Our results demonstrated that corticospinal excitability was not altered by LV and similar results were observed for intracortical excitability. Those results were observed whether responses to single- or double-pulse TMS, respectively, were measured during isometric contraction or at rest.

In the present study, corticospinal excitability remained unchanged after prolonged LV, both when measured at rest and during isometric contraction. This first result has already been demonstrated during isometric contraction performed after 30 min of LV^14,16,17^ as well as when corticospinal excitability was investigated on relaxed muscles after prolonged LV^32,33^. Corticospinal excitability can be influenced by cortical and spinal excitability and previous studies reported a decrease in spinal excitability after prolonged LV suggesting an increase in cortical excitability^14,16,17^. This increase in cortical excitability could be to the result of increased descending drive during the low-intensity contraction to maintain the same level of contraction, compensating for the decrease in spinal excitability. However, this would not explain previous findings of increased resting corticospinal excitability 30–60 min after prolonged LV^32,33^. Therefore, an alternative hypothesis to explain previous findings could be an increased intrinsic cortical excitability after LV due to repeated Ia afferents discharges targeting cortical sensorimotor areas^34^. This may rely on topographically and functionally specific reciprocal connections between primary somatosensory cortex and primary motor cortex^32^. As such, LV can be considered as a proprioceptive intervention that can induce synaptic reorganisation responsible for long-term potentiation and cortical plasticity^4^.

**Figure 6:**
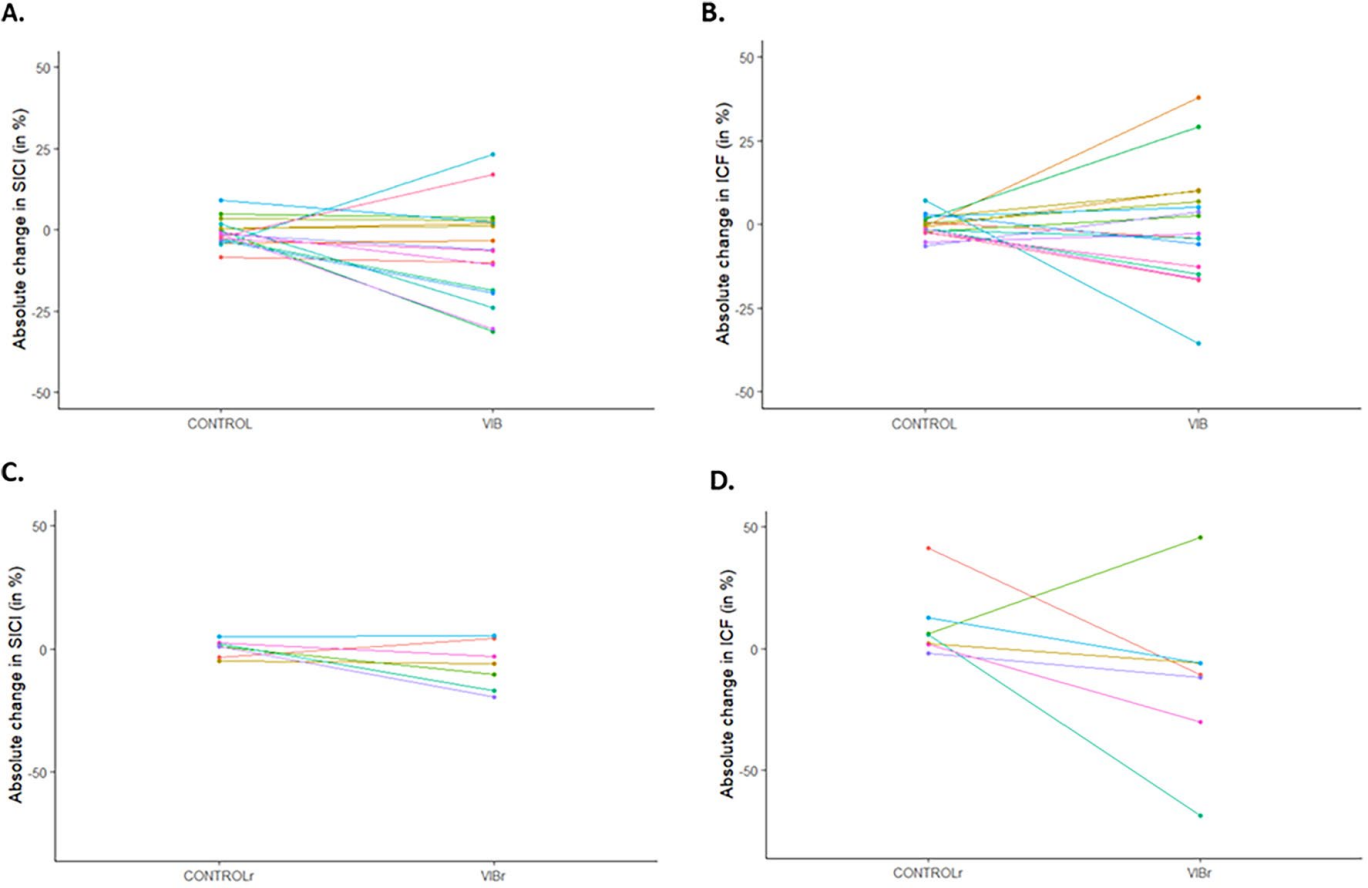
Individual differences in absolute change in SICI (POST–PRE) for CONTROL and VIB conditions (panel **A**), absolute change in ICF (POST–PRE) for CONTROL and VIB conditions (panel **B**), as well as absolute change in SICI (POST–PRE) for CONTROLr and VIBr conditions (panel **C**) and absolute change in ICF (POST–PRE) for CONTROLr and VIBr conditions (panel **D**). Positive values indicate more inhibition (SICI) or facilitation (ICF) for POST than PRE measurements and negative values indicate less inhibition or facilitation. Participants are displayed with the same colour across panels (**A**–**D**).

Some studies previously investigated intracortical excitability after prolonged LV. While a decrease in SICI, together with an increase in ICF, was anecdotally reported^18^, other studies demonstrated an absence of SICI^9,10,19^, long-interval intracortical inhibition (LICI)^9^ or short-interval intracortical facilitation (SICF)^19^ modulation immediately after LV exposure (note that in the study of Miyara et al.^19^, decreased SICI was however observed when measured 30 min after the end of FV intervention, and that in the study of Rosenkranz and Rothwell^9^, changes in SICI after prolonged LV were actually observed when measurements were performed during ongoing LV, contrasting with what was observed in the no-vibration condition). Yet measurements were performed at rest, what may be suboptimal considering recent EEG findings suggesting that an increase in cortical activity after prolonged LV is only observed when recordings are performed during a voluntary contraction^21^. For instance, there is during muscle contraction a disinhibition of corticospinal neurons with a decrease in SICI^35^ and an increase in ICF^36^ when compared to rest. Accordingly, cortical integration of Ia afferents inputs has been reported to be facilitated during muscle contraction^37^, leading us to speculate that intracortical excitability measurements would be more sensitive to prolonged LV if performed during actual muscle contraction than at rest. Yet we did not observe any significant modulation of SICI nor ICF after prolonged LV when measured during muscle contraction, and this was similarly observed when measurements were performed at rest. Regarding SICI, discrepancies between our results, together with similar findings of previous studies^9,10,19^, and those of Christova et al.^18^ showing vibration-induced changes in SICI remain however to be further understood. The same holds true when considering absence of ICF modulation in the present study compared to an increase in Christova et al.^18^ study. When considering PRE-to-POST changes in both CONTROL and VIB conditions, we however observed a greater inter-individual variability for the VIB condition (Fig. 6). And this was observed both when measurements were performed during muscle contraction and at rest. This may suggest that the prolonged LV exposure actually had an effect on intracortical excitability, yet with dissimilarities between participants.

Variability of responses to a given intervention has already been described in exercise training with some individuals considered as no responders, showing little to no improvement, whereas responders significantly improved after a specific training regime^38^. Responders and non-responders to LV have also been suggested after LV training^2^. Moreover, in a study investigating the acute effect of prolonged LV exposure on sensorimotor integration^39^ demonstrated increased conditioned MEP amplitude (i.e. conditioning being obtained through peripheral electrical stimulation) only for some participants (i.e. responders). In the present study, while we cannot rule out the possibility that the increased variability observed for the vibration condition when compared to the control condition may be due to variability of the measurements^40^, this may also suggest the presence of responders and non-responders to the LV intervention. Although the latter affirmation remains purely speculative, there is a combination of genetic and environmental components that predispose some individuals to be more responsive than others to a given intervention, as shown as an example for VO2max trainability^41^. Moreover, attention paid to stimulation by the participants has been suggested as a factor potentially influencing individual responses to prolonged sensory stimulation^9,42,43^, a factor we did not control in the present study. Then, we cannot rule out the possibility that attention paid to LV by our participants differed, leading to the observed dissimilarities in their acute changes in intracortical excitability. Further studies are therefore needed to determine to what extent attentional processes may play a role in the observed results and to what extent there may exist responders and non-responders to prolonged LV exposure. The latter could be investigated by repeating the control and vibration interventions several times for the same participants in order to attribute, or not, such variability to the LV intervention^38^. For instance, we remind that our interpretation here is speculative, especially because potentials responders presented opposite directions for their changes in intracortical excitability (i.e. some decreased SICI or ICF while others increased). Moreover, when attempting subgroup analysis, we were unable to visually identify a clear trend of consistent responses to LV for our potential responders between SICI and ICF (e.g.,, a given participant can present decreased SICI and unchanged ICF while another one can present decreased SICI together with increased ICF) (see supplementary data, Fig. 1).

### Limitations

The current investigation is subject to certain limitations that should be noted. First, our approach was exploratory rather than confirmatory and we did not conduct a power calculation as the true data generating model was unknown. We therefore couldn’t determine the required sample size a priori and we cannot rule out the possibility that the statistical power of our study is limited. Moreover, regarding the chosen interstimulus interval (ISI), it has previously been demonstrated no inhibition at an ISI of 2 ms, likely because such ISI could lead to occlusion of inhibitory effects produced by the conditioning pulse^44^. We however opted for a 2-ms ISI as it was the one eliciting the greatest inhibition in our pilot testing, and because this interval has also been consistently reported to exhibit a pronounced peak of inhibition in other studies^28,30,45^. Regarding ICF paradigm, we acknowledge that almost half of our participants were without facilitation in baseline conditions (Fig. 5). It has been accordingly reported that ICF have a high variability with number of trials where participants may show no facilitation^46–49^. This has been proposed to potentially result from interindividual variability for optimal conditioning stimulus intensity to induce ICF^50^. Increasing conditioning stimulus intensity in the present study would likely have enhanced facilitation^47^. Another solution would have been to increase the number of conditioning pulses^50^. Moreover we did not track alterations in spinal excitability during our investigation, despite spinal/motoneuronal excitability is known to be depressed after prolonged LV^2,51^. While it is difficult to speculate on how such vibration-induced changes in spinal/motoneuronal excitability could influence the results of the present study, incorporating measurements of spinal excitability together with measurements of intracortical excitability would be an intriguing avenue for further research, as it could further help better understand the inter-individual variability we observed.

Furthermore, we did not conduct repeated measurements of rMT or aMT after each experimental condition, an approach that was performed by Christova et al.^18^. This decision was influenced by previous research suggesting a lack of modulation in motor thresholds following a period of vibration^18,25^. Consequently, we aimed to minimize stimulation duration and focus on the acute effects of vibration, though this choice may be considered a limitation of our study since we cannot fully rule out the possibility that motor threshold could have been changed by the vibration intervention in the present study, therefore influencing our outcomes. Moreover, we cannot rule out the possibility that an insignificant vibration-induced change in rMT could significantly influence intracortical excitability measurements as well. Lastly, we should acknowledge that our rMT measurements presented significant differences between CONTROLr and VIBr conditions (Supplementary data; Table 1). Yet the mean coefficient of variation that can be calculated from those data is 4.2% while the ICC is 0.96, those values being in the range of what has been previously reported in reliability studies^52^. While it remains difficult to explain such intra-individual variability, we assume this is due to inherent variability of the measurement, through either random sources or ‘noise’ in the technique despite experimental precaution^46^.

## Conclusion

In summary, our findings indicate that intracortical excitability, whether assessed during isometric contraction or at rest, did not exhibit significant changes after prolonged LV exposure. However, it is worth noting that we observed an intriguing rise in inter-individual variability following LV exposure. This may suggest the possibility of divergent responses to prolonged LV exposure among participants, distinguishing between individuals who respond and those who do not. To gain a comprehensive understanding of the extent of responders and non-responders to prolonged LV exposure, and the reasons behind those differential behaviours, additional investigations are warranted.

### Supplementary Information

Supplementary Figure 1.

Supplementary Table 1.

Supplementary Table 2.

## Data Availability

The datasets used and analysed during the current study are available from the corresponding author on reasonable request.
