# Supplementary material for: Effects of prolonged vibration to the flexor carpi radialis muscle on intracortical excitability
Source: Sci Rep. 2024 Apr 11;14:8475. doi: 10.1038/s41598-024-59255-5 (PMC11009410; doi:10.1038/s41598-024-59255-5)
Supplement: Supplementary file 1 — Supplementary Figure 1. [file 41598_2024_59255_MOESM1_ESM.docx]

**Supplementary Figure 1**


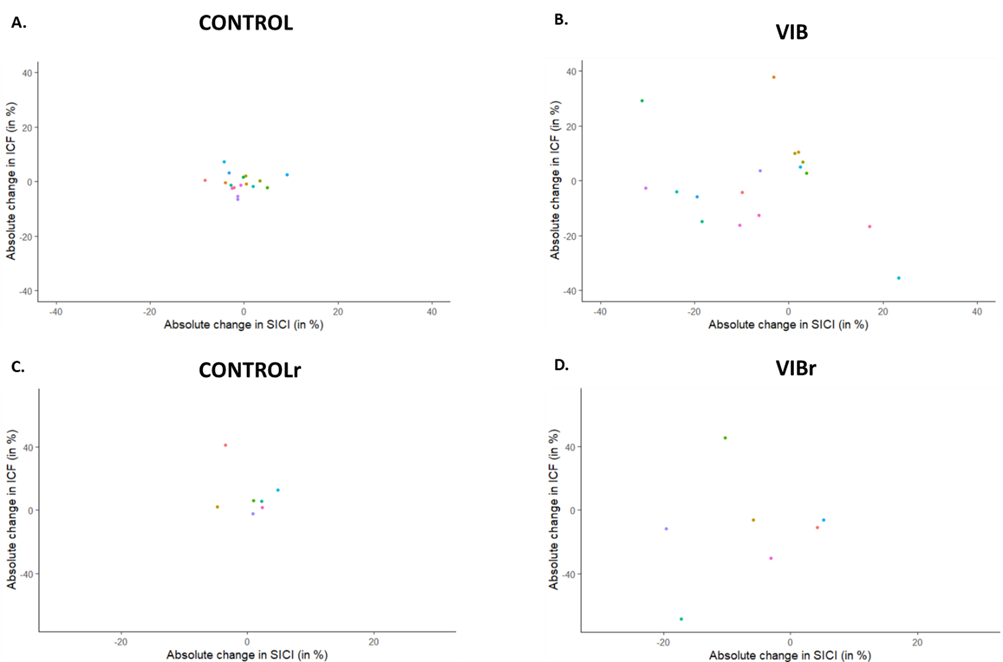


**Supplementary** **Figure 1.** Graphs plotting individual absolute changes (POST-PRE) in both SICI and ICF for CONTROL (panel A), VIB (panel B), CONTROLr (panel C), and VIBr (panel D). Positive values indicate more inhibition (SICI) or facilitation (ICF) for POST than PRE measurements and negative values indicate less inhibition or facilitation. Participants are displayed with the same colour across panels A, B, C and D.
