# Supplementary material for: Effects of prolonged vibration to the flexor carpi radialis muscle on intracortical excitability
Source: Sci Rep. 2024 Apr 11;14:8475. doi: 10.1038/s41598-024-59255-5 (PMC11009410; doi:10.1038/s41598-024-59255-5)
Supplement: Supplementary file 2 — Supplementary Table 1. [file 41598_2024_59255_MOESM2_ESM.docx]

**Supplementary table 1**

| **Subjects** | **CONTROLr** | | | **VIBr** | | | **CONTROL** | | | **VIB** | | |
| --- | --- | --- | --- | --- | --- | --- | --- | --- | --- | --- | --- | --- |
|  | *70% rMT* | *rMT* | *120% rMT* | *70% rMT* | *rMT* | *120% rMT* | *70% aMT* | *aMT* | *120% aMT* | *70% aMT* | *aMT* | *120% aMT* |
| 1 | 39 | 56 | 67 | 44 | 63 | 76 | 33 | 47 | 56 | 32 | 45 | 54 |
| 2 | 34 | 49 | 59 | 39 | 56 | 67 | 29 | 41 | 49 | 36 | 52 | 62 |
| 3 | 39 | 55 | 66 | 40 | 57 | 68 | 39 | 55 | 66 | 43 | 62 | 74 |
| 4 | 42 | 60 | 72 | 44 | 63 | 76 | 34 | 49 | 59 | 39 | 55 | 66 |
| 5 | 34 | 48 | 58 | 34 | 48 | 58 | 29 | 42 | 50 | 29 | 42 | 50 |
| 6 | 29 | 41 | 49 | 29 | 42 | 50 | 25 | 35 | 42 | 24 | 34 | 41 |
| 7 | 25 | 36 | 43 | 27 | 38 | 46 | 28 | 40 | 48 | 27 | 39 | 47 |
| 8 |  |  |  |  |  |  | 18 | 26 | 31 | 22 | 31 | 37 |
| 9 |  |  |  |  |  |  | 27 | 39 | 47 | 22 | 31 | 37 |
| 10 |  |  |  |  |  |  | 36 | 52 | 62 | 38 | 54 | 65 |
| 11 |  |  |  |  |  |  | 24 | 34 | 41 | 23 | 33 | 40 |
| 12 |  |  |  |  |  |  | 34 | 49 | 59 | 31 | 44 | 53 |
| 13 |  |  |  |  |  |  | 22 | 32 | 38 | 29 | 42 | 50 |
| 14 |  |  |  |  |  |  | 22 | 31 | 37 | 29 | 41 | 49 |
| 15 |  |  |  |  |  |  | 23 | 33 | 40 | 22 | 32 | 38 |
| 16 |  |  |  |  |  |  | 25 | 35 | 42 | 26 | 37 | 44 |
| 17 |  |  |  |  |  |  | 29 | 42 | 50 | 28 | 40 | 48 |
| ***MEAN*** | **35** | **49** | **59** | **37** | **52** | **63** | **28** | **40** | **48** | **29** | **42** | **50** |
| ***SD*** | **6** | **9** | **10** | **7** | **10** | **12** | **6** | **8** | **10** | **6** | **9** | **11** |

**Supplementary Table 1**. Individual as well as mean ± SD values of motor threshold stimulation intensities (in %MSO) at rest (rMT) for resting conditions (CONTROLr and VIBr) and during a 10% MVC contraction (aMT) for contraction conditions (CONTROL and VIB). Stimulation intensities used for paired stimulations (i.e. 70 and 120% of rMT or aMT) are further presented. Note that threshold values are significantly different between CONTROLr and VIBr conditions for rMT (p=0.025) but not aMT (p=0.176).
