# Supplementary material for: Effects of prolonged vibration to the flexor carpi radialis muscle on intracortical excitability
Source: Sci Rep. 2024 Apr 11;14:8475. doi: 10.1038/s41598-024-59255-5 (PMC11009410; doi:10.1038/s41598-024-59255-5)
Supplement: Supplementary file 3 — Supplementary Table 2. [file 41598_2024_59255_MOESM3_ESM.docx]

**Supplementary table 2**

| **Subjects** | **CONTROLr** | | **VIBr** | | **CONTROL** | | **VIB** | |
| --- | --- | --- | --- | --- | --- | --- | --- | --- |
|  | PRE | POST | PRE | POST | PRE | POST | PRE | POST |
| 1 | 0.3 | 0.2 | 0.3 | 0.2 | 0.5 | 0.6 | 0.6 | 0.5 |
| 2 | 0.4 | 0.4 | 0.4 | 0.3 | 1.1 | 1.1 | 1.7 | 2.0 |
| 3 | 0.7 | 0.8 | 0.3 | 0.7 | 1.2 | 1.0 | 1.7 | 1.4 |
| 4 | 0.7 | 0.3 | 0.5 | 0.5 | 0.9 | 0.9 | 0.7 | 0.6 |
| 5 | 1.4 | 1.7 | 1.3 | 1.3 | 1.2 | 1.0 | 1.8 | 2.2 |
| 6 | 0.4 | 0.3 | 0.4 | 0.6 | 1.6 | 1.4 | 0.5 | 0.7 |
| 7 | 0.2 | 0.2 | 0.1 | 0.1 | 1.1 | 0.9 | 1.3 | 1.2 |
| 8 |  |  |  |  | 0.7 | 0.6 | 0.5 | 0.5 |
| 9 |  |  |  |  | 0.9 | 1.0 | 1.1 | 0.4 |
| 10 |  |  |  |  | 0.9 | 0.9 | 0.7 | 1.3 |
| 11 |  |  |  |  | 1.8 | 1.6 | 2.1 | 1.6 |
| 12 |  |  |  |  | 1.0 | 1.1 | 0.9 | 0.9 |
| 13 |  |  |  |  | 0.8 | 0.9 | 1.3 | 1.3 |
| 14 |  |  |  |  | 0.6 | 0.6 | 0.6 | 0.5 |
| 15 |  |  |  |  | 1.9 | 2.3 | 2.4 | 0.9 |
| 16 |  |  |  |  | 2.6 | 2.5 | 2.5 | 2.0 |
| 17 |  |  |  |  | 1.2 | 1.2 | 1.5 | 1.5 |
| **MEAN** | **0.6** | **0.6** | **0.5** | **0.5** | **1.2** | **1.1** | **1.3** | **1.1** |
| **SD** | **0.4** | **0.6** | **0.4** | **0.4** | **0.5** | **0.5** | **0.7** | **0.6** |

**Supplementary Table 2**. Individual as well as mean ± SD values of test (i.e. non-conditioned) motor evoked potentials (in mV) before (PRE) and after (POST) resting (CONTROLr and VIBr) and contraction (CONTROL and VIB) conditions. No significant differences were observed between conditions and time points.
